# Abortion Changes Among Residents of an Abortion Rights Protective State

**DOI:** 10.1001/jamanetworkopen.2024.60460

**Published:** 2025-02-18

**Authors:** Kelly A. DeBie, Margaret J. Gutilla, Kayleigh P. Keller, Jennifer L. Peel, Andreas M. Neophytou

**Affiliations:** 1 Department of Environmental and Radiological Health Sciences, Colorado State University, Fort Collins; 2 Colorado School of Public Health, Colorado State University, Fort Collins; 3 Department of Health and Exercise Science, Colorado State University, Fort Collins; 4 Department of Statistics, Colorado State University, Fort Collins

## Abstract

This cross-sectional study examines whether passage of a 6-week abortion ban in Texas in 2021 was associated with changes in abortion rates among Colorado residents.

## Introduction

There have been substantial changes in abortion access at the state and national level. Texas state legislators passed SB8 in 2021, enacting a 6-week gestational limit.^1^ Access to safe, legal abortion is necessary for comprehensive health care.^2^ Although research has focused on residents of states enacting restrictions, less is known about changes for residents of states where abortion access remains legal. Colorado is a full abortion access state with no gestational limitations.^3^ Leveraging data on abortion procedures performed in Colorado, we sought to determine whether SB8 in Texas was associated with changes in abortion for Colorado residents.

## Methods

 This cross-sectional study was deemed exempt by the Colorado State University institutional review board, and informed consent was not needed because no individual patient data were used, in accordance with 45 CFR §46. We adhered to the STROBE reporting guidelines. We analyzed monthly abortion count data from January 2018 to June 2024 from the Colorado Department of Public Health and Environment, including information on residence state and gestational week at the time of the abortion. Generalized linear quasi-Poisson regression interrupted time series models were used to compare rates before and after changes in the law, adjusting for both long-term time trends and seasonality using September 2021 as the interruption point. We assumed no confounders beyond time and selected a study design well-suited to analyze policy change using repeated observations of data. No lag-to-effect was included given the time-limited nature inherent in pregnancy. All analysis for this study was conducted using R statistical software version 4.3.1 (R Project for Statistical Computing); preliminary data cleaning was performed using Excel 365 (Microsoft). Statistical significance was evaluated at the α = .05 level for 2-sided tests.

## Results 

**Figure 1.**
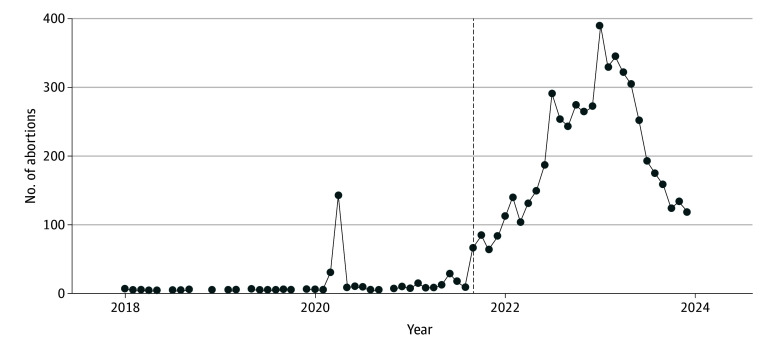
Raw Monthly Abortion Counts Among Texas Residents Seeking Abortions in Colorado Vertical line indicates implementation date of Texas SB8.

**Figure 2.**
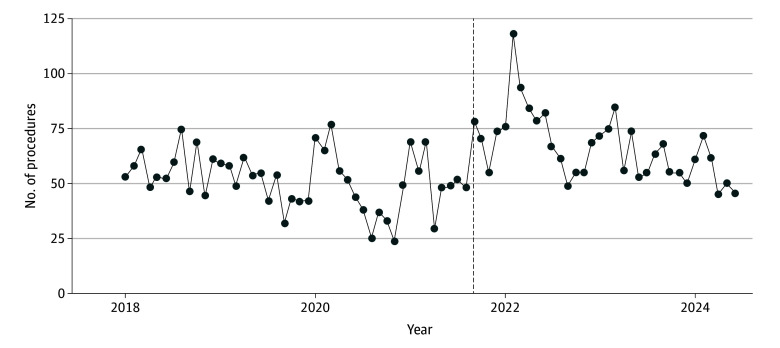
Raw Monthly Second Trimester Abortion Counts Among Colorado Residents Vertical line indicates implementation date of Texas SB8.

The percentage of abortions provided in Colorado to out-of-state residents increased from 13% in 2020 to 30% in 2023. Figure 1 shows raw data for Texas residents traveling to Colorado for abortions and indicates a clear increase after the implementation of SB8. Figure 2 visualizes the raw data for Colorado residents using the same interruption point, showing an increase in second trimester procedures temporally matching the demand for procedures from Texas residents, peaking 6 months after SB8. Adjusting for time trends, regression results show that compared with the time before Texas SB8, Colorado residents were overall 11% more likely to have procedures in the first trimester (rate ratio, 1.11; 95% CI, 1.00-1.24; *P* = .04) and 83% more likely to have procedures in the second trimester (rate ratio, 1.83; 95% CI, 1.55-2.17; *P* < .001) after SB8.

## Discussion

In this cross-sectional study, we found a statistically significant increase in abortions for Colorado residents in association with the implementation of Texas SB8. This aligns with reports from area clinicians who experienced dramatic increases in patient demand after SB8, also reported by the media, resulting in delayed appointments for everyone.^4^ Second trimester findings concur with the work of Dindinger et al,^5^ who previously studied changes in gestational age at the time of abortion in Colorado through the end of 2022 in association with SB8. Potential consequences of delayed procedures may include increased cost, increased complexity of the procedure, the emotional toll of waiting, and the potential for having the pregnancy revealed. Although travel of Texas residents to Colorado for abortions has declined, it has not returned to baseline. The increase found in second trimester abortions for Colorado residents appears to have resolved, offering reassurance of health care capacity to adjust to increased demand. Expanded access to telehealth services, increased self-managed abortion, or care in other protective states may also be alleviating strain on the system.^6^ Strengths of this study include the use of monthly data in the selected design. The possibility of unmeasured confounding, the available data representing a likely undercounting of abortions, and residence misclassification are potential limitations of these findings. We also recognize that these data can capture information only for those able to travel and do not include individuals without the money, support, and resources to do so.

## Notes

Supplement.
Data Sharing Statement
