# Supplementary material for: Abortion Changes Among Residents of an Abortion Rights Protective State
Source: JAMA Netw Open. 2025 Feb 18;8(2):e2460460. doi: 10.1001/jamanetworkopen.2024.60460 (PMC11836752; doi:10.1001/jamanetworkopen.2024.60460)
Supplement: Supplement. — Data Sharing Statement [file jamanetwopen-e2460460-s001.pdf]

Data Sharing Statement

DeBie. Abortion Changes Among Residents of an Abortion Rights Protective State. *JAMA Netw Open*. Published February 18, 2025. doi:10.1001/jamanetworkopen.2024.60460

Data
Data available: Yes
Data types: Deidentified participant data, Data dictionary
How to access data: Aggregated data and data dictionary will be available upon request at Dryad repository. https://doi.org/10.5061/dryad.dz08kps64
When available: With publication

Supporting Documents
Document types: None

Additional Information
Who can access the data: Anyone requesting the data
Types of analyses: For any purpose
Mechanisms of data availability: With investigator support
